# Genomic characterization of multidrug-resistant *Escherichia coli* strains identified from patients with urinary tract infection in Egypt

**DOI:** 10.1038/s41598-026-40536-0

**Published:** 2026-03-11

**Authors:** Nancy M. El Halfawy, Mona K. Gouda, Fatma A. Elgayar, Alaa Aboelnour Badran

**Affiliations:** 1 Department of Botany and Microbiology, Faculty of Science, Alexandria University, Alexandria, Egypt; 2 Department of Clinical Pathology, Faculty of Medicine, Mansoura University, Mansoura, Egypt

**Keywords:** *Escherichia coli*, Extended-spectrum β-lactamases (ESBLs), Urinary tract infection (UTI), Horizontal gene transfer (HGT), Computational biology and bioinformatics, Genetics, Microbiology

## Abstract

Extended-spectrum β-lactamases-producing *Escherichia coli* (ESBL-EC) pose a serious threat. Moreover, widespread antimicrobial use in Egypt increased the prevalence of antimicrobial resistance (AMR). In this study, whole-genome sequencing (WGS) using the Illumina NovaSeq 6000 was performed on two isolates (UPE7 and UPE139) recovered from participants with urinary tract infections to characterize their resistomes and virulomes. Antibiotic resistance and virulence genes of the two clinical *E. coli* strains were predicted using computational analysis tools. Several virulence traits and antibiotic resistance genes (ARGs) were identified. Strain UPE7 harbored *bla*_TEM−1B_, *bla*_CTM−X−15,_ *bla*_CMY−2_, and strain UPE139 revealed the presence of *bla*_OXA−244_, *bla*_TEM−12_, *bla*_TEM−82_, and *bla*_CTM−X−15_ rending the resistance phenotype. The presence of mobile genetic elements adjacent to ARGs thereby suggests their potential for dissemination through horizontal gene transfer. Furthermore, the serotyping in silico investigation revealed that *E. coli* UPE7 and UPE139 serotypes were O8:H9 and O9:H30, respectively. Notably, key mutations in the *gyrA*, *parC*, and *parE* genes were predicted, consistent with their confirmed resistance to levofloxacin. These findings emphasize the importance of genomic surveillance to guide antimicrobial therapy and monitor emerging high-risk clones, and they support the need for larger-scale genomic studies to improve epidemiological understanding and clinical relevance.

## Introduction

Antibiotic resistance (AMR) is a major concern in modern healthcare since it possesses the potential to spread worldwide and has limited treatment options^1^. Poverty, poor sanitation, easily available antibiotics without prescription, and ignorant clinical practice are factors that aid in the spread of multidrug-resistant (MDR) microbial strains in community and hospital settings^2^. In addition, antibiotics are extensively employed in agriculture to enhance crop yields and in animal husbandry, particularly in cattle farming, where they are administered for therapeutic, prophylactic, and metaphylactic purposes, as well as at controlled concentrations in animal feed as growth promoters to prevent disease and safeguard animal health^3,4^. Therefore, the extensive use of antibiotics outside human medicine has accelerated the emergence and dissemination of MDR bacteria, representing an escalating threat to both animal and public health^4^. New examples of AMR are regularly reported, and the time needed for bacteria to become resistant to newly introduced antibiotics is getting shorter^5^.

*Escherichia coli* was listed among the World Health Organization (WHO) priority organisms that cause critical infections and require the formulation of new treatments^6^. Remarkably, the resistance of *E. coli* strains to third-generation antibiotics is mainly associated with the production of extended-spectrum β-lactamases (ESBLs) globally^7^. In the last decade, ESBL-EC infections have become challenging because of the rapid pandemic spread of antimicrobial-resistant clones and limited treatment options that put the patient’s life in danger^8,9^. Furthermore, ESBL-EC is often linked to an increased incidence of nosocomial infections in hospitals^10^. *E. coli* isolates may carry multiple antibiotic resistance genes carried on mobile genetic elements (MGEs), including plasmids, transposons, integrons, and insertion sequences (IS), which can spread to compatible neighboring species via horizontal gene transfer (HGT)^11,12^. In addition, *E. coli* harbors diverse virulence factors (adhesion-associated fimbriae, hemolysin, siderophores, flagella, capsular polysaccharides, and outer membrane proteins) that contribute to distinct aspects of pathogenicity^13^. The combined action of these determinants enables the bacterium to establish infection, persist with the host, and evade defense mechanisms^14^.

Whole genome sequencing (WGS) has emerged as a feasible approach for detecting antibiotic resistance. Indeed, comprehensive genetic analysis via next-generation sequencing (NGS) enables high-resolution genotyping of clinically significant MDR strains and facilitates targeted antibiotic usage directly guided by genetic sequence^15^. In addition, NGS can study the evolutionary relationship of MDR strains from different geographical regions. Moreover, it is a helpful tool for tracking MGEs, identifying emerging resistance mechanisms, and uncovering chromosomal mutations^10,16^.

In this study, two MDR *E. coli* strains, UPE7 and UPE139, were isolated from patients with urinary tract infections (UTIs) at Mansoura University Hospital (Egypt). To characterize the two uropathogenic *E. coli* strains in-depth, WGS using Illumina sequencing was conducted. Additionally, MLST, serotyping, and phylotyping were used to investigate the clonal relatedness of the isolates. Genome assemblies of UPE7 and UPE139 were screened for the presence of ARGs, MGEs, and virulence factors on the genomic level.

## Results

### Antimicrobial resistance profile

The two isolates, UPE7 and UPE139, were identified from patients with UTI in Egypt and revealed extensive resistance patterns **(**Table 1**)**. Among aminoglycosides, both isolates exhibited high-level resistance to gentamicin (≥ 32 µg/mL for UPE7; ≥16 µg/mL for UPE139) and amikacin (≥ 64 µg/mL for both). UPE7 and UPE139 strains were resistant to penicillins and β-lactams, including ampicillin-sulbactam, cefoperazone-sulbactam, and piperacillin-tazobactam, as well as extended-spectrum cephalosporins such as ceftazidime, cefotaxime, and cefepime. Carbapenems, including ertapenem, imipenem, and meropenem, also demonstrated elevated MIC values, confirming carbapenem resistance in both isolates. Resistance extended to fluoroquinolones, with high MIC values reported to levofloxacin (≥ 16 µg/mL for UPE7; ≥8 µg/mL for UPE139). Additionally, strain UPE139 revealed intermediate resistance to chloramphenicol, whereas polymyxin B exhibited resistance in UPE7 (8 µg/mL) but intermediate resistance in UPE139. Both strains were resistant to the sulfonamide antibiotic, trimethoprim-sulfamethoxazole.

**Table 1:** Minimal inhibitory concentrations (MICs) interpretation of *Escherichia coli* UPE7 and UPE139 as per CLSI 2021 guidelines.

Antibiotics	MIC values (µg/mL)	
	UPE7	UPE139
Gentamicin	≥ 32 **(R)**	≥ 16 **(R)**
Amikacin	≥ 64 **(R)**	≥ 64 **(R)**
Ampicillin-sulbactam	≥ 64/32 **(R)**	≥ 32/16 **(R)**
Ceftazidime-clavulanate	ND	≥ 2/8 **(R)**
Cefotaxime-clavulanate	ND	≥ 2/8 **(R)**
Cefoperazone-sulbactam	≥ 128/64 **(R)**	≥ 128/64 **(R)**
Piperacillin-tazobactam	≥ 128/4 **(R)**	≥ 128/4 **(R)**
Ertapenem	≥ 32 **(R)**	≥ 16 **(R)**
Imipenem	≥ 128 **(R)**	= 16 **(R)**
Meropenem	≥ 128 **(R)**	= 8 **(R)**
Cefazolin	ND	≥ 32 **(R)**
Cefuroxime	ND	≥ 64 **(R)**
Cefotaxime	ND	≥ 64 **(R)**
Ceftazidime	≥ 64 **(R)**	≥ 32 **(R)**
Cefepime	≥ 64 **(R)**	≥ 32 **(R)**
Ampicillin	ND	≥ 32 **(R)**
Cefoxitin	ND	≥ 64 **(R)**
Levofloxacin	≥ 16 **(R)**	≥ 8 **(R)**
Azithromycin	ND	≥ 64 **(R)**
Chloramphenicol	ND	= 16 **(I)**
Polymyxin B	= 8 **(R)**	= 1 **(I)**
Minocycline	≥ 32 **(R)**	= 8 **(I)**
Tigecycline	= 8 **(R)**	= 1 **(R)**
Trimethoprim-sulfamethoxazole	≥ 64/1216 **(R)**	≥ 8/152 **(R)**

*R, Resistant; **I**, Intermediate; **ND**, not determined.

### General genomic features of *E. coli* strains UPE7 and UPE139

According to VITEK biochemical identification and genomic characterization, the two isolates were identified as *Escherichia coli*. The predicted genome sizes were 4,828,053 bp and 5,096,432 bp for UPE7 and UPE139, respectively. The G + C contents for both isolates were about 50.6%. Moreover, WGS yielded 681,534 and 992,028 reads for UPE7 and UPE139, respectively. Genomic features, including coding sequence (CDS), rRNA, tRNA, and CRISPR array numbers, are listed in Table 2. The whole genome shotgun projects of UPE7 and UPE139 were submitted to the National Biotechnology Information Centre (NCBI) GenBank database with the accessions **JBHWTZ000000000** and **JBHWUA000000000**, respectively.

**Table 2:** General genomic features of *Escherichia coli* UPE7 and UPE139 genome assemblies.

Assembly metrics	UPE7	UPE139
Length (bp)	4,828,053	5,096,432
GC content (%)	50.61	50.64
CDS	4,764	5,117
tRNA	83	90
rRNA	12	11
CRISPR array	2	3
Plasmids	4	5

### Determination of serotypes, sequence type, and phylogroup

In silico serotyping identified *E. coli* UPE7 as O8:H9, based on the presence of *wzt* and *wzy* alleles (O8) and the *fliC* gene (H9). Similarly, UPE139 was assigned to O9:H30, carrying *wzm* and *wzt* alleles (O9) and the *fliC* gene (H30). Furthermore, MLST allelic analysis was performed based on the allelic combination of seven genes, and the results revealed no clonal relationship between the two isolates assigned to different sequence types. Based on the Pasteur (Achtman) scheme, the sequence type assigned the isolates UPE7 and UPE139 to ST692 (ST410) and ST650 (ST361), respectively. In addition, the Clermont *E. coli* phylotyping assigned UPE7 and UPE139 to phylogroup C (*fdm*, *trpA*,* gpC*, *ybgD*) and phylogroup A (*fdm*, *cfaB*, *ybgD*), respectively.

### Detection of virulence factors

Screening the uropathogenic *E. coli* strains UPE7 and UPE139 genome sequences revealed the occurrence of a diverse array of virulence genes associated with adhesion, host cell invasion, iron acquisition, and persistence in the urinary tract. Strain UPE7 carried genes encoding fimbrial and adhesion factors, including *fimH* (type 1 fimbriae) and *csgA* (curlin major subunit CsgA), *fdeC* (intimin-like adhesion FdeC), *IpfA* (long polar fimbriae), and the *yehABCD* fimbrial cluster, which enhance adhesion and biofilm formation. Additionally, *hlyE* (hemolysin) and *hha* (hemolysin express modulator), *nlpI* (lipoprotein precursor), and *terC* (tellurium ion resistance protein). In comparison, UPE139 harbored several overlapping factors, including *fimH*, *csgA*, *fdeC*, *nlpI*, *hylE*, and the *yehABC* fimbrial cluster, but also possessed additional determinants, *capU* (hexosyltransferase homolog linked to capsule synthesis), *shiA* (associated with the Shigella SHI-2 pathogenicity island), *sitABCD* (iron transport), *tia* (Tia invasion determinant), and *traT* (outer membrane protein complement resistance).

### Detection of antimicrobial resistance genes (ARGs)

**Fig. 1:**
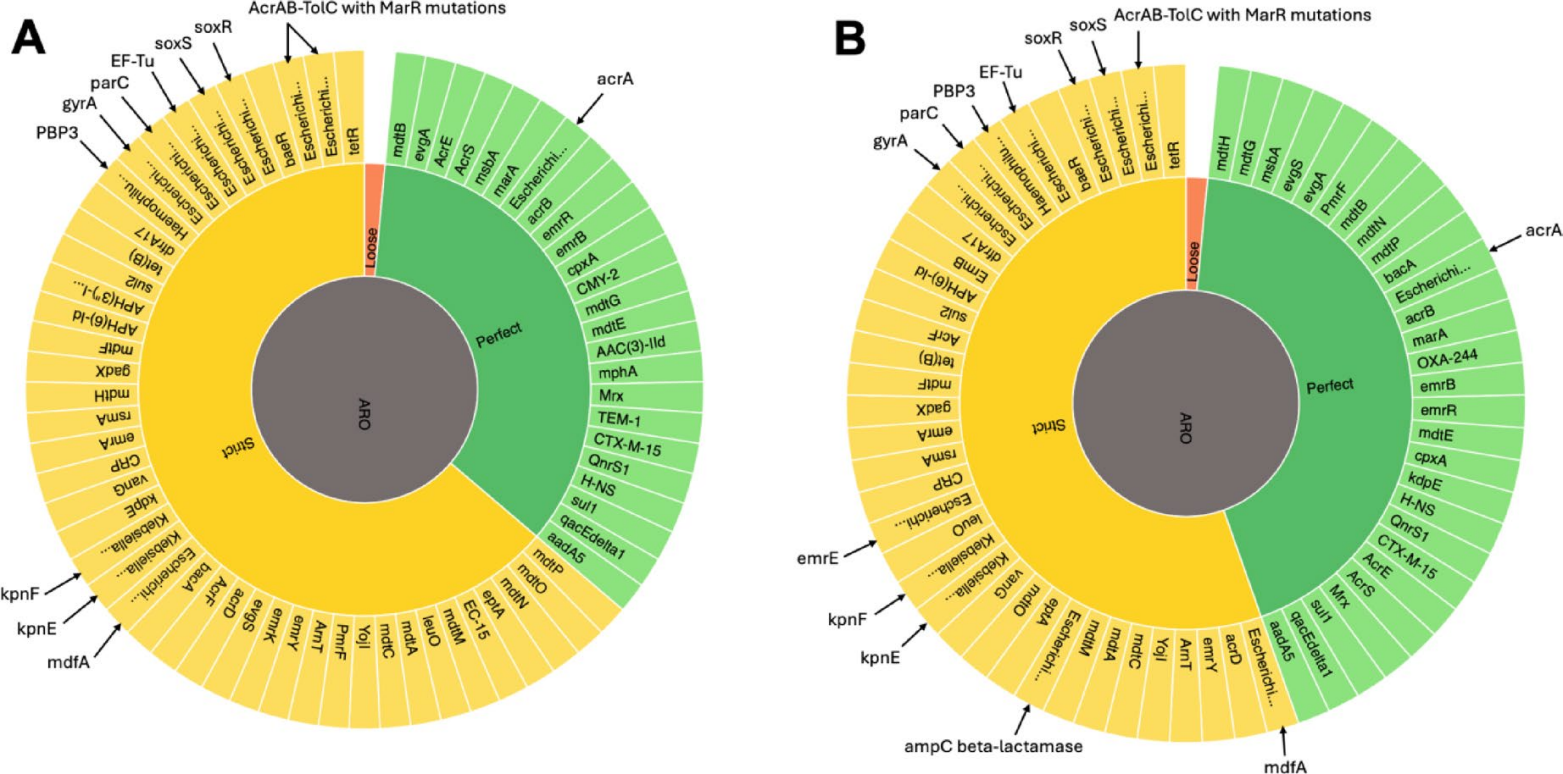
Antimicrobial resistance genes predicted from the whole-genome sequence data of **(A)** *E. coli* UPE7 and **(B)** *E. coli* UPE139 using CARD-RGI (https://card.mcmaster.ca/analyze/rgi). ARO represents the Antibiotic Resistance Ontology, and the surrounding colored segments represent the detection confidence level of AMR genes. Green color (Perfect) indicates AMR genes perfectly matching reference sequences in the database. Yellow color (Strict) indicates that AMR genes are closely related to known AMR, with some variations. Red (Loose) indicated low-confidence matches.

Several ARGs conferring resistance to multiple antibiotic classes were identified in the UPE7 and UPE139 genome sequences, demonstrating a correlation between phenotypic resistance and underlying genetic determinants. The observed resistance to cephalosporins and β-lactams was confirmed by detection of ARGs, including *bla*_TEM−1B_, *bla*_CMY−2_, and *bla*_CTM−X−15_ in the UPE7 isolate, as well as *bla*_TEM−12_, *bla*_TEM−82_, and *bla*_CTM−X−15_ in UPE139. Moreover, high-level aminoglycoside resistance in both isolates was explained by the presence of aminoglycoside genes such as *aph*(3’’)-Ib, *aph*(6)-Id, and *aadA5*. Resistance to tetracyclines, quinolones, macrolides, sulfonamides, and trimethoprim was consistent with the detection of *tetA*, *tetD*, *tetR*, *qnrS1*, *mphA*, *mdfA*, *sul1*, *sul2*, and *dfrA17* in both genome sequences. In addition, the resistance of the UPE139 strain to carbapenems was associated with the occurrence of *bla*_OXA−244_, a member of the OXA-48-like carbapenemase, whereas no carbapenemase resistance genes were detected in the UPE7 genome sequence. In particular, resistance extended to fluoroquinolones is related to mutations in quinolone resistance-determining regions (*gyrA*, *parC*, *parE*,* qnr*). In addition, both strains revealed regulatory and efflux-associated genes such as *acrR*, *mdtK*, and *marR*, which reduce intracellular quinolone concentrations through active efflux. The presence of the *arnABCDTEF* operon in both UPE7 and UPE139 was associated with resistance to polymyxin B (Fig. 1; Table 3).

**Table 3:** Overview of resistome and mobilome in UPE7 and UPE139 strains.

		UPE7	UPE139
**MLST**		ST692^a^ – ST410^b^	ST650^a^ – ST361^b^
**pMLST**		IncF [F1:A1:B49]	IncF [F31:A1:B49]
**Serotype**		O8:H9	O9:H30
**CH type**		4–24 (fumC4, fimH24)	99 − 54 (fumC99, fimH54)
**Antimicrobial Resistance Genes**	**β-lactamase**	*bla*_CMY−2_ *bla*_CTM−X−15_	*bla*_TEM−12_ *bla*_CTM−X−15_
	**Aminoglycoside**	aph(3’’)-Ib aph(6)-Id aac(3)-IIa aac(3)-IId *aadA5*	aph(3’’)-Ib aph(6)-Id *aadA5*
	**Macrolide**	*mphA* *mdfA*	*mphA* *mdfA*
	**Tetracycline**	*tetA* *tetR*	*tetA* *tetD* *tetR*
	**Quinolone**	*qnrS1*	*qnrS1*
	**Polymyxin**	*pmrD* *pmrG* *arnABCDTEF*	*pmrD* *pmrG* *arnABCDTEF*
**Plasmids**		IncFII IncFIA IncFIB Col(BS512)	IncFII IncFIA IncFIB IncQ1 IncY
**Chromosomal mutations**		*gyrA* (S83L, D87N) *parC* (S80I) *parE* (S458A)	*gyrA* (S83L, D87N) *parC* (S80I) *parE* (S458A)

^**a**^**MLST**, Pasteur scheme; ^**b**^**MLST**, Achtman scheme.

**Fig. 2:**
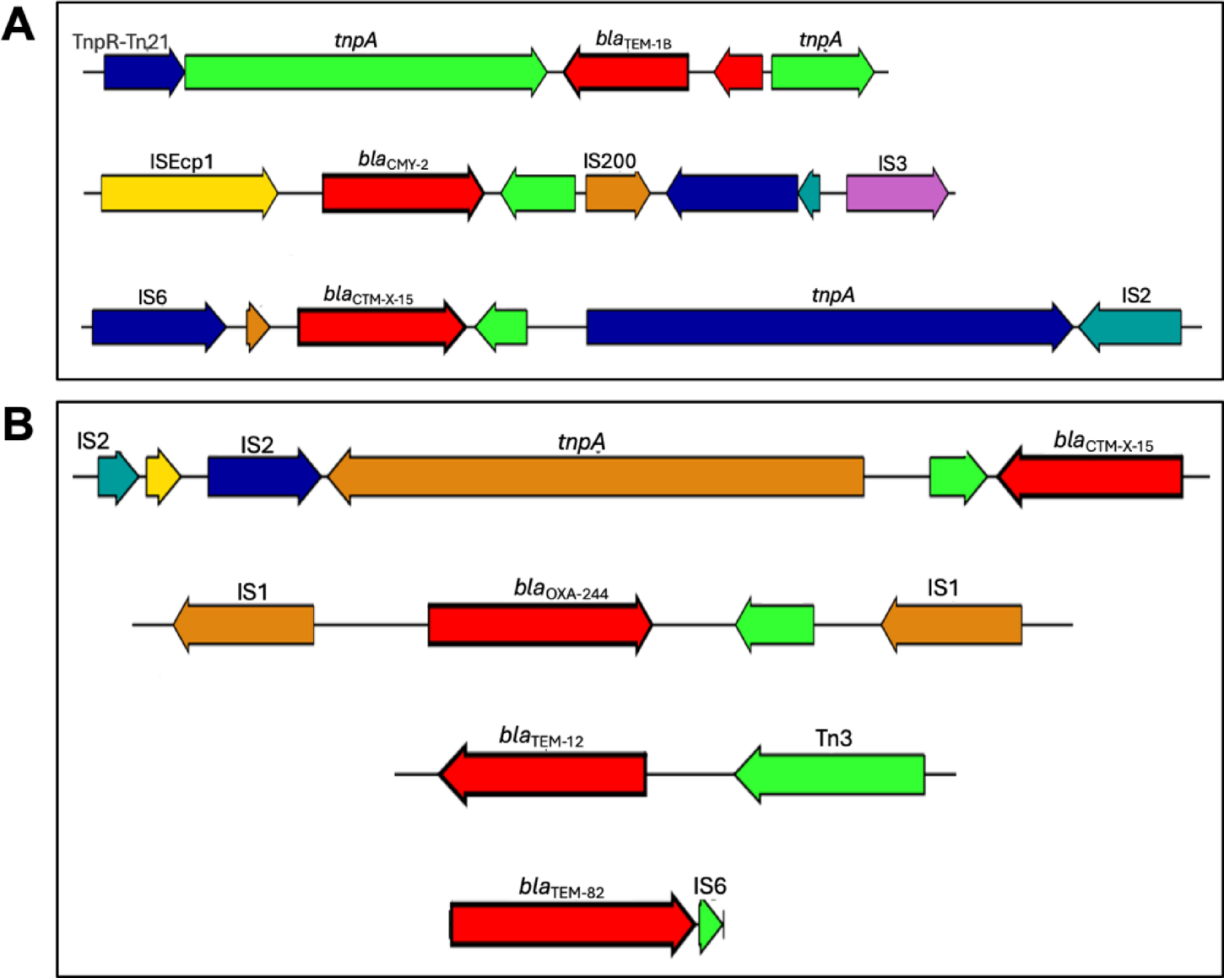
Schematic representation of ARGs and genetic mobile elements detected in **(A)** UPE7 and **(B)** UPE139. The colors represented: ARGs (Red), Transposase-related genes (green), Insertion sequence elements (Blue, Yellow, Orange).

The analysis of the UPE7 genome revealed the presence of ESBL genes near mobile genetic elements, such as insertion sequences and transposons. The investigation revealed that *bla*_TEM−1B_ is located between the TnpA transposon. Moreover, the *bla*_CMY−2_-containing region revealed the occurrence of this gene chromosomally within the cassette comprising IS*Ecp1*-*bla*_CMY−2_-*orf*-IS*200*-*orf*-*orf*-IS*3*. In addition, the *bla*_CTX−M−15_ gene was bracketed by transposon TnpA and the insertion sequence IS*6* **(**Fig. 2A**)**. Meanwhile, the analysis of ESBL-related genes in the UPE139 genome sequence revealed the presence of the *bla*_CTX−M−15_ gene associated with transposon TnpA and IS*2*. Notably, *bla*_OXA−244_ was positioned between two insertion sequences (IS*1*). Moreover, *bla*_TEM−12_ and *bla*_TEM−82_ were found to be associated with transposon Tn3 and insertion sequence IS*6*, respectively **(**Fig. 2B**)**. Therefore, the WGS-based resistance profile confirmed that UPE7 and UPE139 were multidrug-resistant strains.

### Investigating chromosomal mutations

In this study, the UPE7 and UPE139 isolates were phenotypically resistant to levofloxacin, a fluoroquinolone antibiotic. Therefore, the chromosomal mutations in DNA gyrase (*gyrA*) and topoisomerase IV (*parC* and *parE*) were investigated. Notably, two types of *gyrA* mutations (S83L and D87N), one *parC* mutation (S80I), and one *parE* mutation (S458A) were detected in UPE7 and UPE139 genome sequences.

### Prediction of mobile genetic elements (MGEs)

Screening MGEs conferred the presence of numerous genes associated with the mobilization of ARGs in UPE7 and UPE139 strains. *E. coli* UPE7 genome sequence exhibited plasmid replicons, namely Col(BS512), IncFIA, IncFIB (AP001918), IncFII (pAMA1167-NDM-5) and IncQ1. Moreover, the UPE139 genome sequence revealed the presence of plasmid replicons: IncFII, IncFIA, IncFIB, IncQ1, and IncY. Plasmid-mediated quinolone resistance (PMQR) gene *qnrS1* and ESBL gene *bla*_CTX−M−15_ were present in the IncFII plasmid in UPE7 and UPE139 genomes. In addition, several IS and transposable elements were detected in the two strains. UPE7 and UPE139 genome sequences revealed the presence of Insertion sequences IS*Ecp1*, belonging to the IS*1380* family transposase, and IS*200*/IS*605*. Furthermore, IS*3* and IS*6* were found at a relatively high frequency in the genomes of the two isolates.

### Comparative analysis

**Fig. 3:**
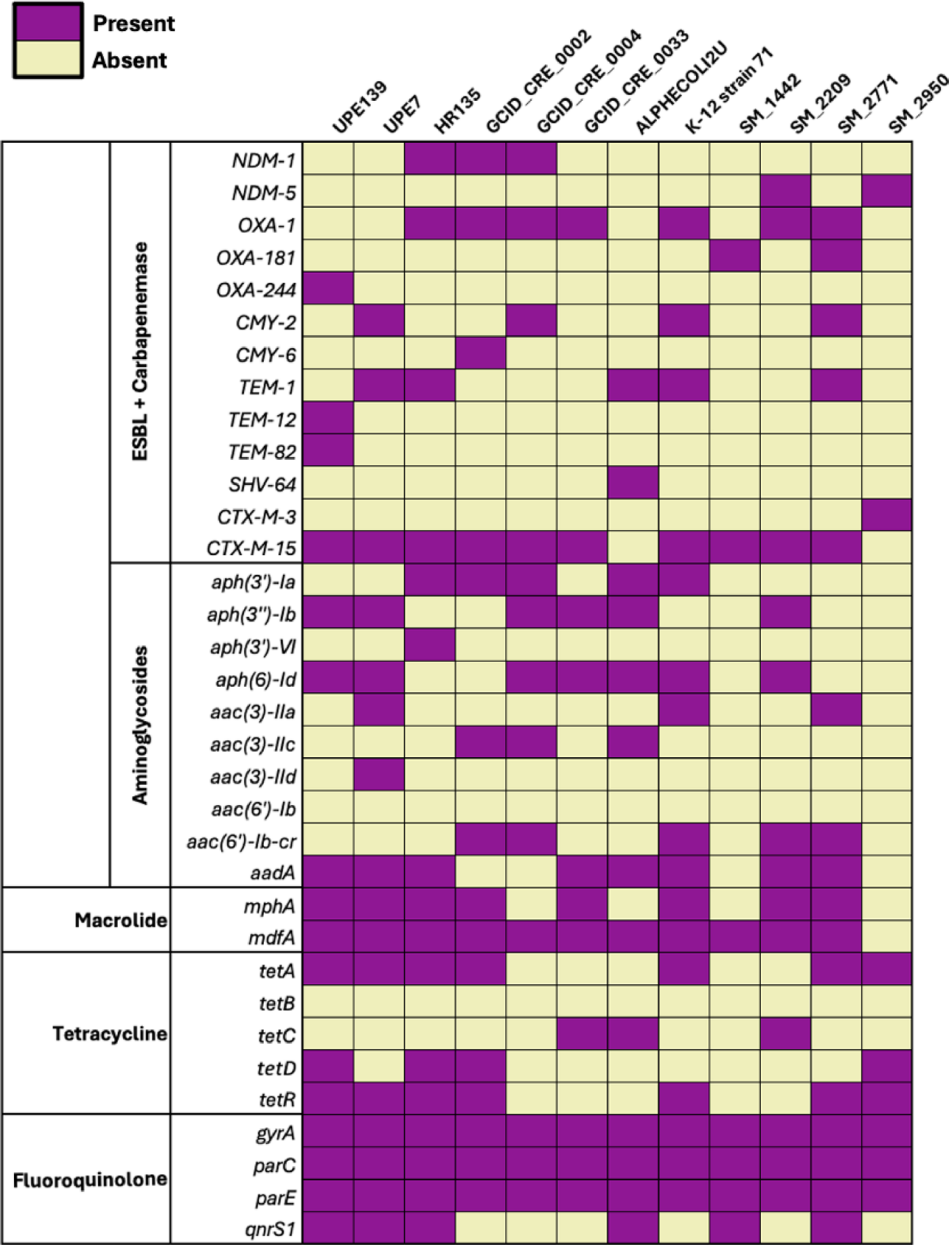
Heatmap represents antibiotic resistance genes in *E. coli* UPE7, *E. coli* UPE139, and ten selected uropathogenic *E. coli* strains using the ARG detection method available through the BV-BRC web server. The purple and yellow colors represent present and absent genes, respectively.

A comparative genomic analysis involving UPE7, UPE139, and ten *E. coli* strains isolated from patients in Egypt was performed **(**Fig. 3**)**. The genomic analyses of the annotated sequenced genomes revealed a variety of phylogroups, serotypes, and distributions of antibiotic resistance genes. The strains demonstrated combinations of ESBL genes, including the most predominant gene *bla*_CTX−M15_, and carbapenem genes such as *bla*_NDM−1_ and *bla*_NDM−5_. Notably, the four strains in this study harbored the *bla*_CMY−2_ gene, and one strain harbored the *bla*_OXA−244_ gene. The studied strains revealed the occurrence of one or more aminoglycoside genes. Most of the investigated strains revealed the presence of the macrolide resistance genes (*mphA* and *mdfA*), which confer macrolide resistance. Moreover, most of the isolates showed quinolone resistance-determining regions (*gyrA*, *parC*, *parE*), as well as the presence of *qnrS1*, which confers plasmid-mediated quinolone resistance.

**Fig. 4:**
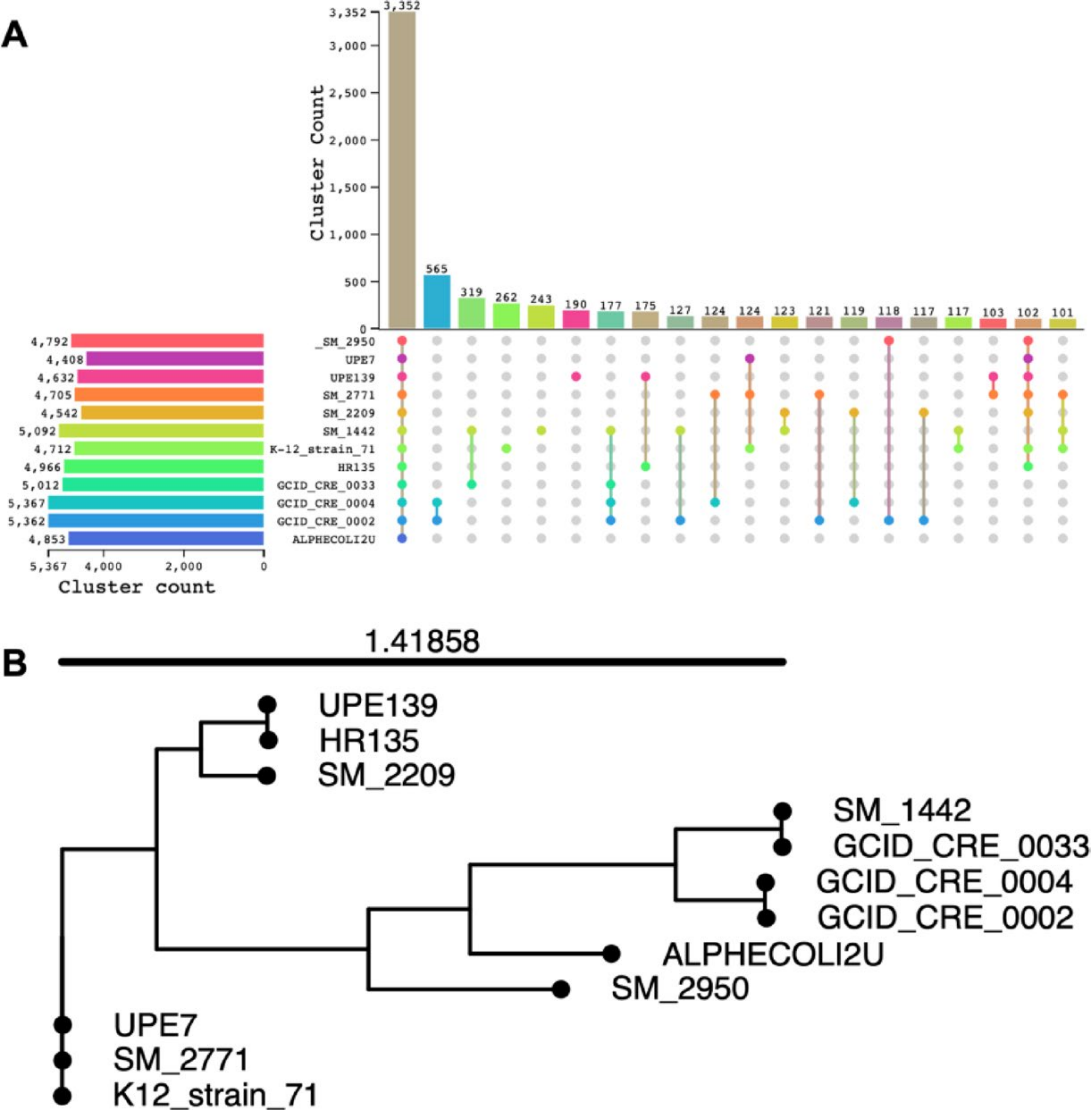
**(A)** Upset figure showing the common genes in UPE7, UPE139, and other selected *E. coli* genomes, indicating the number of shared and unique protein clusters and unique genes. **(B)** Whole genome-based phylogenetic tree of UPE7 and UPE139 isolates, constructed based on the presence/absence of orthologous gene clusters identified using the pan genome analysis tool.

Pan-genome analysis revealed 5,367 orthologous gene clusters identified across the analyzed *E. coli* genome sequences, of which 3,352 shared orthologous gene clusters were identified among the *E. coli* strains under investigation **(**Fig. 4A**)**. Notably, some gene families are involved in critical biological processes. Furthermore, the phylogenetic tree based on whole-genome sequencing revealed the close relation between UPE139 and HR135 (accession number **QXNW00000000**), a phylogroup A strain, which was previously isolated from UTI in Egypt. Moreover, UPE7 was clustered with SM_2771 (accession number **JAJKQA000000000**) and K12 strain 71 (accession number **SSTS00000000**), belonging to phylogroup C and sequence type ST692 **(**Fig. 4B**)**. *E. coli* strains within the same phylogroup displayed varied levels of ARGs.

## Discussion

According to the WHO, ESBL-producing *Enterobacteriaceae* pose a significant risk to human health^17^. Therefore, pathogen surveillance using genomic approaches helps to understand the dynamics, and the results are important for controlling the spread of resistant bacteria^18^. Consequently, identifying resistance traits through WGS is significant in improving diagnosis and innovative regimes for treating patients with UTI^19,20^. In the current study, the resistance genotypes of UPE7 and UPE139 strains corresponded to the phenotypic resistance of antibiotics under investigation, reflecting the correlation between the high resistance rate and the frequent prescription of these antibiotics in the treatment of UTI in Egyptian healthcare^21^.

In Egypt, several findings have demonstrated a higher prevalence of AMR among carbapenem-resistant and ESBL-EC, highlighting the significance of understanding the molecular mechanisms of resistance^22^. The rapid emergence of MDR strains of *E. coli* is a serious concern in developing countries. These high observed rates in cephalosporins and carbapenemes are attributed to antibiotic misuse and extensive usage of third-generation antimicrobials, particularly in Egyptian hospitals^23,24^. Furthermore, various factors, including poor hygiene, antibiotics without prescription, and a lack of rapid diagnostic methods, promote the emergence of these ESBL-producing isolates^25^.

The sequence type UPE7 was ST410, according to Achtman’s scheme. This sequence type was previously identified in *E. coli* EC13655, isolated from a hospitalized patient in Egypt^21^. Furthermore, the uropathogenic *E. coli* NGCE33 strain (ST410 clone) containing an array of β-lactamase genes was previously detected in Bangladesh^26^. *E. coli* of the ST410 lineage is an emerging MDR high-risk clone that has shown an increasing worldwide spread and revealed non-susceptibility to carbapenems, cephalosporins, fluoroquinolones, aminoglycosides, and tetracyclines^27,28^. However, UPE139 was ST361, according to Achtman’s scheme. A previous study revealed that *E. coli* ST361 was a key player in the dissemination of carbapenem resistance in France, due to the chromosomal localization of *bla*_OXA−244_^29^. In addition, the clone ST361 was previously detected in seven patients in Denmark in May 2018^30^. Therefore, it is essential to understand the potential threats of these high-risk clones, use effective antibiotics, and take precautions against their spread.

Clermont phylotyping plays a crucial role in identifying the isolates and developing new therapeutic agents. Phylogenetic group A is usually associated with commensal strains, suggesting that the gastrointestinal tract is the primary source of *E. coli* strains that colonize the urinary tract^31,32^. Most *E. coli* isolates belong to phylogroup A and possess multiple ARGs and fewer virulence determinants^21^. Moreover, phylogroup C possesses a high rate of MDR *E. coli* with lower motility^33^.

Investigating the virulome of *E. coli* strains UPE7 and UPE139 more in depth through WGS revealed a diverse set of virulence determinants that underline their pathogenic potential and ability to persist in the urinary tract environment. Both strains carried fimbrial and adhesion-related genes, which are strongly associated with biofilm formation^34^. The presence of *sitABCD*, an iron uptake system that acquires iron to boost their pathogenicity potential, tends to restrict iron bioavailability, a traditional host defense mechanism against bacterial invasion^35^. Moreover, the presence of hemolysin genes in both isolates is associated with disease severity in UTI and indicates the cytotoxic activity against a broad range of cells^36^. The identification of *shiA*, typically associated with the Shigella SHI-2 pathogenicity island, points toward horizontal gene transfer events that may have expanded UPE139’s virulence repertoire^37^. These findings suggest that while both UPE7 and UPE139 exhibit core uropathogenic-associated virulence traits, UPE139 appears to possess a more complex virulence profile, which may confer increased adaptability and pathogenic potential in the urinary tract.

CTX-M-type ESBLs producing *E. coli* are associated with the international dissemination of MDR high-risk clones^38^. Several studies have revealed that *bla*_CTX−M−15_, one of the most prevalent genotypes, is widely found in *E. coli* and rapidly disseminates within patients with urinary tract infections^39,40^. A previous study reported that *bla*_CTX−M−15_ was predominant and present in 68.4% of urinary ESBL-producing *E. coli* isolates in Minia University Hospital (Minia, Egypt)^41^. In addition, the location of the *bla*_CTX−M−15_ gene adjacent to IS*26* (which belongs to the IS*6* family) might be involved in their mobilization^42^. Over the past two decades, CMY-2-like enzymes have been the most commonly observed in ESBL-EC^43^. In this study, the *bla*_CMY−2_ gene was flanked by a single copy of insertion sequence IS*Ecp1*, which facilitates mobilization of the adjacent ARG sequence by a one-end transposition mechanism^21^. Studies have reported the occurrence of CMY-2 in *E. coli* (strain UEC59) isolated from urinary tract infection patients in Pakistan, and *E. coli* isolates in Mexico^44,45^. The *bla*_CMY−2_ gene was recently predicted in 10% of tested *E. coli* strains isolated from hospitalized patients in Alexandria Main University Hospital (Alexandria, Egypt)^21^.

OXA-244 carbapenemase was detected in the *E. coli* UPE139 genome sequence. It differs from OXA-48 by a single amino acid substitution, showing reduced activity against carbapenem antibiotics^46^. A previous study reported the presence of *bla*_OXA−244_ in the genome of MDR *E. coli* strain HR14_AS, recovered in December 2014 from a wound pus swab of a male patient in Egypt^47^. Similar to our results, *bla*_OXA−244_ is chromosomally integrated into an ISR1-related Tn6237 transposon, which is responsible for the dissemination of OXA-244-producing *E. coli*^48^. Despite the absence of known carbapenemase-encoding genes in the genome sequence of UPE7, the isolate revealed resistance to carbapenems. This result is in agreement with a previous study, which reported that imipenem-resistant *E. coli* ECV219 did not carry genes associated with carbapenem resistance^49^. This suggests the involvement of an alternative resistance mechanism, including inducible overexpression of chromosomal cephalosporinases, such as AmpC (e.g., CMY-2), combined with porin loss that can result in clinically significant carbapenem resistance^50,51^.

Healthcare-associated infections caused by isolates with *mcr* genes that contribute to colistin resistance are associated with increased morbidity and mortality rates, as these plasmid-borne genes facilitate horizontal transfer between bacterial species, promoting their rapid global dissemination^24^. In this study, although the genome sequences of UPE7 and UPE139 were *mcr*-negative, both isolates exhibited phenotypic resistance to polymyxin. This indicates that polymyxin B resistance is mediated by chromosomally encoded mechanisms. Polymyxins belong to a group of cationic antimicrobial peptides, and they owe their antimicrobial action to binding to lipid A, a component of the negatively charged lipopolysaccharide (LPS) in the outer membrane of Gram-negative strains, leading to cell lysis^52,53^. Moreover, resistance to colistin in *E. coli* can be acquired through chromosomal point mutation, particularly in genes encoding two-component regulatory systems, such as PhoPQ and PmrAB^54,55^. These mutations result in the expression of the *arnBCDTEF* operon, which leads to lipopolysaccharide modification via the addition of 4-amino-4-deoxy-l-arabinose (l-Ara4N) to lipid A, thereby reducing the net negative charge of the outer membrane and conferring resistance to polymyxins in *E. coli*^56^.

Notably, *gyrA* (S83L, D87N), *parC* (S80I), and *parE* (S458A) mutations were reported in UPE7 and UPE139 isolates. Fluoroquinolone resistance is mainly caused by point mutations in genes encoding DNA gyrase and topoisomerase IV, such as *gyrA*, *gyrB*, *parC*, and *parE* in quinolone resistance-determining regions^57^. These types of mutation are associated with high resistance levels to fluoroquinolones in MDR *E. coli* strains^58^. In addition, resistance to fluoroquinolones is dependent on the capacity of bacteria to reduce drug accumulation through different efflux systems^59^. A previous study in Thailand reported that *E. coli* urinary isolates have a high percentage of resistance against levofloxacin, greater than 90%^60^. Moreover, a recent study conducted in Egypt demonstrated that 58.7% of isolates of Gram-negative clinical pathogens have at least one quinolone resistance (*qnr*) gene^61^.

Studying MGEs is significant in understanding and suggesting the mobility dynamics of ARGs among pathogenic strains. The isolates UPE7 and UPE139 genome sequences harbor multiple plasmid replicons. Previous studies have denoted that IncF plasmids are associated with highly drug-resistant properties and may likely cause long-term resistance and pathogenicity in patients and hospital environments^10^. This agreed with a previous study from Egypt that demonstrated the presence of IncF replicon types in MDR *E. coli* isolates^62^. Similar to our results, previous studies have shown that plasmids expressing the ESBL phenotype also carry genes encoding resistance to antibiotics such as tetracyclines, fluoroquinolones, and aminoglycosides^63,64^. In agreement with a previous study, the Tn3 transposon harbors the β-lactamase TEM gene, which is transmitted between plasmids of different replicon types^39^.

This study was limited to only two *E. coli* isolates, which restricts the extent to which the findings can be generalized to the broader population of uropathogenic *E. coli* strains in Egypt. Although these isolates were selected based on distinct antimicrobial resistance profiles and prior epidemiological relevance, they do not encompass the full genetic and phenotypic diversity present in clinical settings. To address these limitations, future studies should include a larger and more representative collection of isolates, combined with complementary phenotypic and transcriptomic analyses, to provide a more comprehensive understanding of resistance mechanisms and their clinical implications.

## Methods

### Sample collection and characterization

A hundred clinical specimens were collected from females whose ages ranged from 20 to 40 years in a tertiary hospital (Mansoura, Egypt). The study was approved by the University Institutional Review Board of Mansoura University **(IRB: R.25.01.3002)**. Ethical considerations, including personal data privacy, were considered during all study steps. Isolation was performed on MacConkey agar plates (MAC; SRL, India), followed by bacterial purification and conventional identification using the VITEK 2 system (BioMérieux, France) available in the Clinical Pathology Department, Faculty of Medicine. Pure cultures were stored at −20 °C in tryptic soy broth (TSB; SRL, India) supplemented with 50% (v/v) glycerol for further phenotypic and genotypic characterization.

### Determination of minimum inhibitory concentration (MIC)

The minimum inhibitory concentration of the isolates was determined using the DL microbial AST system (Zhuhai DL Biotech, China; https://en.medicaldl.com). The results were obtained after 24 h against 24 antimicrobial agents, including gentamicin, amikacin, ampicillin-sulbactam, ceftazidime-clavulanate, cefotaxime-clavulanate, cefoperazone-sulbactam, piperacillin-tazobactam, ertapenem, imipenem, meropenem, cefazolin, cefuroxime, cefotaxime, ceftazidime, cefepime, ampicillin, cefoxitin, levofloxacin, azithromycin, chloramphenicol, polymyxin B, minocycline, tigecycline, and trimethoprim-sulfamethoxazole. The minimum inhibitory concentrations (MIC, µg/mL) of the isolates were interpreted as susceptible (S), intermediate (I), or resistant (R) according to Clinical and Laboratory Standards Institute (CLSI) guidelines^65^. UPE7 and UPE139 strains were categorized phenotypically as MDR and positive for ESBL based on the resistance profiles. Thus, the two strains were subjected to a comprehensive genomic investigation.

### Whole-genome sequencing, assembly, and functional annotation

Genomic DNA from *E. coli* UPE7 and UPE139 was extracted using the GeneJET™ Genomic DNA Purification Kit (Thermo Fisher Scientific, UK) following the manufacturer’s instructions. Library preparation was carried out on a Hamilton Microlab STAR automated liquid handling system (Hamilton Bonaduz AG, Switzerland) using the Nextera XT Library Prep Kit (Illumina, UK). Genome sequencing was outsourced by MicrobesNG (Birmingham, UK; http://microbesng.uk), on the Illumina NovaSeq 6000 platform (Illumina, USA) using a 250 bp paired-end protocol with 30X sequence coverage. The sequencing adaptors and low-quality reads were trimmed using Trimmomatic (Version 0.30) with a sliding cut-off of Q15^66^. Subsequently, *de novo* genome assembly was performed using SPAdes software (Version 3.7.0)^67^. The newly sequenced genomes were annotated using Prokka software (Version 1.11) and the nucleotide Basic Local Alignment Search Tool (BLASTn) available through the NCBI Prokaryotic Genome Annotation Pipeline (PGAP)^68,69^.

### Determination of serotypes, sequence type (MLST), and phylogroup

The serotypes of *E. coli* strains were determined with SeroTypeFinder 2.0 provided by the Centre for Genomic Epidemiology (CGE; https://cge.cbs.dtu.dk/services/SerotypeFinder/) for the identification of somatic (O) and flagellar (H) antigens. Multi-locus sequence type (MLST) was determined using the Institute Pasteur web server (https://bigsdb.pasteur.fr)^70^. The phylogroups of UPE7 and UPE139 were determined using Clermont typing^71^.

### Identification of virulence, mutations, and antimicrobial resistance genes

Virulence factors in *E. coli* UPE7 and UPE139 genome sequences were investigated using the VirulenceFinder-2.0 server (https://cge.food.dtu.dk/services/VirulenceFinder/), available through CGE. Antimicrobial resistance genes (ARGs) were identified in the UPE7 and UPE139 genome sequences using the Bacterial and Viral Bioinformatics Resource Centre (BV-BRC; https://www.bv-brc.org/) webserver (Version 3.39.10)^72^ and Comprehensive Antibiotic Resistance Database (CARD-RGI; https://card.mcmaster.ca/) with 90% identity and ≥ 60% coverage thresholds^73^. Chromosomal mutations mediating AMR were analyzed using the ResFinder 4.6.0 server^74^ available through CGE.

### Identification of plasmids and insertion sequences (IS)

The genome sequences of the two *E. coli* strains were investigated for plasmids using the PlasmidFinder (Version 2.0) online tool^75^. Plasmids of the IncF type were subtyped by the plasmid MLST webserver (https://pubmlst.org/organisms/plasmid-mlst). Insertion sequences (IS) were determined using ISfinder (https://www-is.biotoul.fr) using BLASTn (Version 2.2.31+)^76^.

### Comparative genomic analyses

A total of ten genome sequences associated with urinary tract infections were selected based on their geographic relevance to Egypt, and the sequences were retrieved from the NCBI database **(**Table 4**)**. Furthermore, comparative genomics analysis was performed using OrthoVenn3 (https://orthovenn3.bioinfotoolkits.net) and the Integrated Prokaryotes Genome and Pan-Genome Analysis service (IPGA Version 1.09; https://nmdc.cn/ipga/)^77,78^. A whole genome-based phylogenetic tree of UPE7 and UPE139 isolates, and other *E. coli* strains, was created using the IPGA web server^78^.

**Table 4:** *Escherichia coli* strains were used in this study, including the GenBank accession number.

Strain	Accession Number	Geographical location	Isolation Source	Year	tRNA	rRNA	GC %	^a^MLST	^b^Phylogroup
UPE7	JBHWTZ000000000	Egypt	Urine Infection	2022	83	12	50.61	ST692	C
UPE139	JBHWUA000000000	Egypt	Urine Infection	2022	90	11	50.64	ST650	A
HR135	QXNW00000000	Egypt	Urine Infection	2016	84	8	50.41	ST650	A
GCID_CRE_0002	RYXW00000000	Egypt	Urine	2018	76	4	50.54	ST1244	B2
GCID_CRE_0004	RYXV00000000	Egypt	Urine Infection	2018	75	4	50.50	ST1244	B2
GCID_CRE_00033	RYXG00000000	Egypt	Urine	2018	71	4	50.78	ST43	B2
SM_1442	JAJKSZ000000000	Egypt	Urine	2015	83	22	50.67	ST43	B2
SM_2209	JAJKRT000000000	Egypt	Urine	2015	70	4	50.75	ST2	A
SM_2771	JAJKQA000000000	Egypt	Urine	2016	72	3	50.50	ST692	C
SM_2950	JAJKPP000000000	Egypt	Urine	2016	81	6	50.50	ST44	D
ALPHECOLI2U	JAENHQ000000000	Egypt	Urinary Catheter	2019	84	9	50.51	ST641	G
K-12 strain 71	SSTS00000000	Egypt	Urine	2019	65	1	50.55	ST692	C

^**a**^**MLST**, Pasteur scheme; ^**b**^**Phylogroup**, Clermont typing.

## Conclusions

This study offers insights into the prevalence of antibiotic resistance mechanisms and mobile genetic elements in two *E. coli* strains (UPE7 and UPE139) isolated from patients with urinary tract infections in Egypt. The clinical isolates were identified as multidrug-resistant, exhibiting resistance to multiple classes of antimicrobials. Genomic analysis of MGEs highlighted their role in the rapid dissemination of MDR traits across different clonal types of pathogenic *E. coli*. These findings underscore the importance of combining phenotypic testing with genomic approaches to guide the treatment of urinary tract infections and inform effective control strategies. However, the limited number of isolates analyzed, future studies involving larger and more diverse collections of clinical strains are essential to validate these results and capture broader resistance patterns. Moreover, subsequent investigations will include plasmid sequencing to further explore the presence and transferability of resistance genes. Additionally, expanded genomic surveillance across different geographic regions and healthcare settings will be critical for tracking the emergence and dissemination of high-risk clones, identifying resistance genes, and guiding antibiotic stewardship efforts.

## Data Availability

The whole genome sequences of **E. coli** UPE7 and **E. coli** UPE139 were deposited at the DDBJ/ENA/GenBank database under the accession numbers JBHWTZ000000000 and JBHWUA000000000, respectively.
